# Multiple evanescent white dot syndromes

**DOI:** 10.1007/s12348-011-0051-9

**Published:** 2011-11-26

**Authors:** Ruwan A. Silva, Thomas A. Albini, Harry W. Flynn

**Affiliations:** Department of Ophthalmology, Bascom Palmer Eye Institute, Miller School of Medicine, University of Miami, 900 NW 17th Street, Miami, FL 33136 USA

**Keywords:** MEWDS, Inflammation, Spectral-domain OCT, White dot syndrome

## Abstract

**Purpose:**

The aim of this study is to report a patient with multipe evanescent white dot syndrome (MEWDS) presenting with classic foveal granularity and pathology localized to the outer retina.

**Methods:**

Case study methodology was used in the current study.

**Results:**

A 34-year-old Caucasian female presented with photopsias and blurry vision in her left eye. Examination, particularly the foveal granularity noted in her affected eye, was archetypal for the diagnosis of MEWDS. Fundus autofluorescence, fluorescein and indocyanine green angiography were also consistent with this diagnosis. Spectral-domain optical coherence tomography (SD-OCT) demonstrated increased retinal pigment epithelium granularity and disruption of the photoreceptor inner segment–outer segment junction subfoveally.

**Conclusions:**

Foveal granularity may be the most specific feature of MEWDS with SD-OCT capable of localizing pathology to the outer retina—a historically controversial finding.

## Introduction

Multiple evanescent white dot syndrome (MEWDS) was first described in 1984 as a unilateral disturbance in visual acuity notable for white dots of the “the retinal pigment epithelium (RPE) or deep retina and a granularity of the fovea” [1]. Associated clinical features include flu-like prodrome, predisposition to young females, blurred disc margins, and temporal scotomata. Of note is that even in its original description, attention was paid to the fluorescein angiography (FA) pattern demonstrating the now characteristic early hyperfluorescence and late staining which “seemed to be due to RPE alterations” [1]. It was these RPE changes, based both on the FA pattern and clinical appearance of the disease, which led to the early hypothesis that damage to the RPE was the “initial pathologic event” in this disease [2]. Since that time, much controversy has emerged over both the initial disease site as well as etiology. We here report a case of MEWDS notable for both its classic retinal appearance, foveal granularity as well as delineating the photoreceptor region of the neurosensory retina as the principal ocular site affected by this disease.

## Case report

**Fig. 1:**
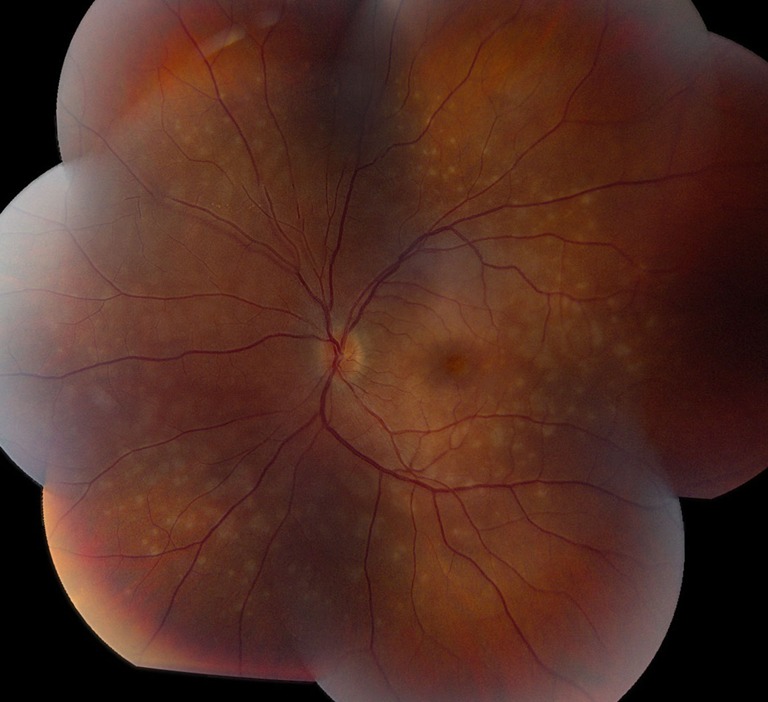
Fundus photo of the left eye demonstrating optic nerve head edema, foveal granularity, and a circinate distribution of retinal pigment epithelium (RPE) white dots

**Fig. 2:**
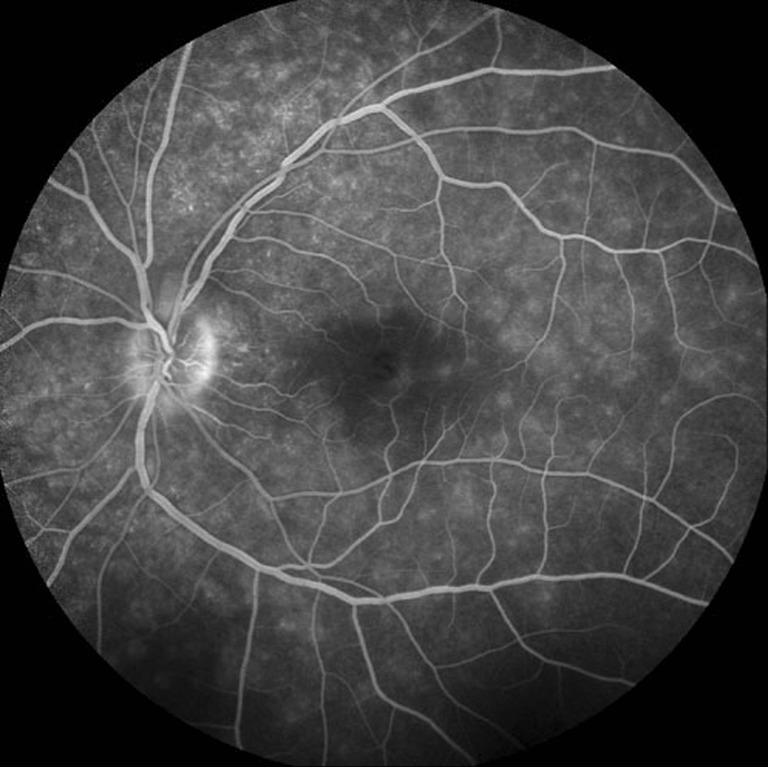
Late-phase fluorescein angiography demonstrating early RPE staining in a parafoveal distribution as well as late leakage around the optic nerve

**Fig. 3:**
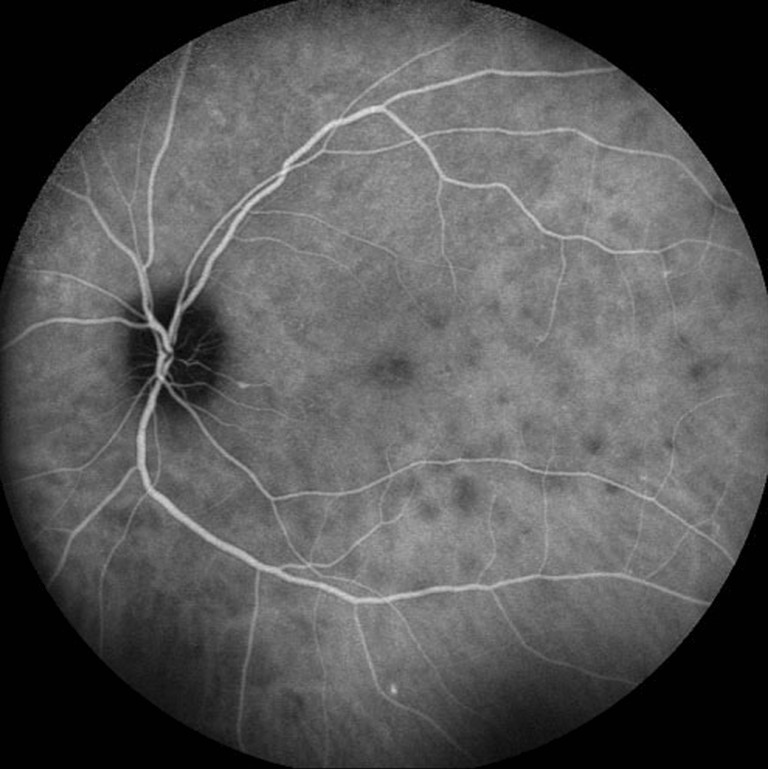
Late-phase indocyanine green angiography (corresponding to Fig. 2) demonstrating punctuate late hypofluorescence

**Fig. 4:**
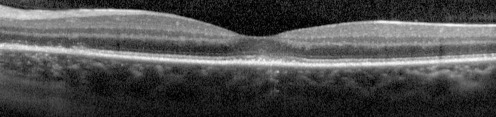
Spectral-domain optical coherence tomography (SD-OCT) demonstrating disruption of the photoreceptor inner segment–outer segment (IS–OS) junction subfoveally with preservation of the external limiting membrane

A 34-year-old Caucasian female with a past ocular history significant for ocular migraines presented with a 5-day history of blurry vision in her left eye with occasional photopsias. Her initial visual acuity was 20/20 in the right eye and 20/30 in the left eye. Anterior segment examination was significant for trace anterior chamber cell. Posterior segment examination (Fig. 1) demonstrated trace vitreous cell, grade II optic nerve head edema, foveal granularity, and a circinate distribution of RPE white dots. Fundus autofluorescence demonstrated hyperautofluorescence in the distribution corresponding to altered RPE pigmentation. Fluorescein angiography demonstrated early hyperfluorescence with late RPE staining in a parafoveal distribution as well as late leakage around the optic nerve (Fig. 2). Corresponding indocyanine green angiography demonstrated punctuate late hypofluorescence (Fig. 3). Finally, spectral-domain optical coherence tomography (SD-OCT) demonstrated disruption of the subfoveal photoreceptor inner segment–outer segment (IS–OS) junction with possible RPE perturbation (Fig. 4). The latter is suggested mainly by three subfoveal findings: increased RPE granularity, enhanced signal penetration into the underlying choroid, and hyper-reflectivity just internal to the RPE (Fig. 4). The patient was diagnosed with MEWDS with a plan to observe her clinical course without ophthalmic intervention.

## Discussion

When first described in 1984, MEWDS was characterized electrophysiologically by an acute decline in the amplitude of both the early receptor potential (ERP) and electroretinogram (ERG) a-wave with a prolonged ERP regeneration time [2]. Thus, even from this very first report, MEWDS had been prominently linked to photoreceptors as the first known disease involving profound electrophysiologic impairment of the photoreceptor outer segments with full recovery to normal. The specific deficit in photoreceptors was hypothesized to involve either a decrease in the quantity of photoreceptor pigment or misalignment of photoreceptors as implied by the observed ERP [2], either of which may account for the foveal granularity most specific to the disease. The electrophysiology findings, though clearly implying dysfunction of the photoreceptor layer, were initially deemed secondary consequences of (primary) RPE dysfunction. It is likely that the FA pattern in MEWDS contributed to this interpretation as the characteristic hyperfluorescent lesions were seen as window defects thus localizing MEWDS principally to the RPE.

With the widespread clinical use indocyanine green angiography (ICGA) in the 1990s, MEWDS came to be seen as a choroidopathy. Specifically, the observation that early-phase ICGA showed no abnormalities discredited the FA-based conception of RPE window defects (which would have predicted early ICGA hyperfluorescent lesions corresponding to those seen on FA) [3, 4]. The additional observation of late-phase hypofluorescent lesions on ICGA which outnumbered those seen on FA gave rise to the notion that MEWDS involved either choroidal ischemia or exudative choroidal lesions into which indocyanine green (ICG) could not enter [3, 5]. It was further suggested that only a subset of these choroidal lesions perturbed the RPE and outer retina function sufficiently to account for the characteristic white dots of the RPE and hyperfluorescent lesions observed on FA [3, 5]. Our case suggests a component of choroidal ischemia or inflammation (the distinction of which is not presently feasible) in demonstrating hypofluorescent lesions which correspond to a subset of hyperflourescent FA lesions (Fig. 3 **,** inferior arterial arcade). The choroid, however, is unlikely the primary site of pathology as the numerous lesions seen on FA (i.e., Fig. 2, proximal superior arterial arcade) lack a correlate on ICG (Fig. 3 **)**.

With advances in imaging, MEWDS is now understood to be an outer retinal disease. The advent of ultrahigh-resolution optical coherence tomography capable of 3 μm axial resolution has clearly identified pathologic disruptions of the IS/OS junction in cases of MEWDS without evidence of either RPE disturbances (which would be predicted by acute choroidal ischemia) or photoreceptor cell body loss [4, 6]. Localization of pathology to the photoreceptor layer is further supported by electrophysiology analysis noting multifocal ERG and full-field ERG amplitudes corresponding with IS/OS line integrity in the acute and recovery phase of MEWDS [4]. Quantitative measurements of photoreceptor length have even measured photoreceptor outer segment shortening during the acute phase of the disease with gradual relengthening paralleling visual recovery [4, 7]. It has also been noted that the preservation of other retinal layers, most notably the outer nuclear layer, perhaps accounts for the potential for full visual recovery after photoreceptor outer segment disruption unique to MEWDS [7]. We here present a case of MEWDS both classic in appearance on posterior segment photography and angiography with SD-OCT studies clearly demonstrating disruption of the IS–OS junction with preservation of other outer retinal layers.
